# Erratum: Rose et al. When in Need of an ESCRT: The Nature of Virus Assembly Sites Suggests Mechanistic Parallels between Nuclear Virus Egress and Retroviral Budding. *Viruses* 2021, *13*, 1138

**DOI:** 10.3390/v13091705

**Published:** 2021-08-27

**Authors:** Kevin M. Rose, Stephanie J. Spada, Vanessa M. Hirsch, Fadila Bouamr

**Affiliations:** 1 Department of Molecular and Cell Biology, California Institute for Quantitative Biosciences, University of California—Berkeley, Berkeley, CA 94720, USA; 2 Laboratory of Molecular Microbiology, National Institute of Allergy and Infectious Diseases, National Institutes of Health, Bethesda, Rockville, MD 20894, USA; stephanie.spada@nih.gov (S.J.S.); vhirsch@niaid.nih.gov (V.M.H.); bouamrf@mail.nih.gov (F.B.)

The authors wish to make the following erratum to this paper [1]:

The published version of Figure 1 was missing labels. The correct Figure 1 is listed below:

The list of authors was incorrect and did not accurately reflect the contributions of individuals that contributed to the overall work. It is changed to:

Kevin M. Rose ^1,^*, Stephanie J. Spada ^2^, Vanessa M. Hirsch ^2^ and Fadila Bouamr ^2^

^1^ Department of Molecular and Cell Biology, California Institute for Quantitative Biosciences, University of California—Berkeley, Berkeley, CA 94720, USA

^2^ Laboratory of Molecular Microbiology, National Institute of Allergy and Infectious Diseases, National Institutes of Health, Bethesda, Rockville, MD 20894, USA; stephanie.spada@nih.gov (S.J.S.); vhirsch@niaid.nih.gov (V.M.H.); bouamrf@mail.nih.gov (F.B.)

* Correspondence: kevin_rose@berkeley.edu

The Author Contribution section was changed to: S.J.S. conceptualized nuclear envelope mechanisms. F.B. co-wrote the manuscript. K.M.R., V.M.H.–writing. All authors have read and agreed to the published version of the manuscript.

The authors would like to apologize for any inconvenience caused to the readers by these changes.

## Figures and Tables

**Figure 1:**
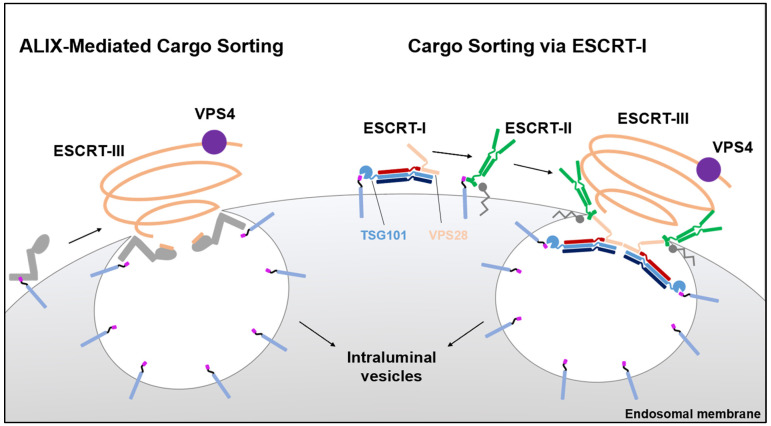
The respective roles of ALIX and ESCRT-I in the sorting of membranous cargo. Upon internalization, ubiquitylated cargo is detected by ALIX (**left**) and ESCRT-I (**right**) for compartmentalization into intraluminal vesicles that are destined for degradation via the late endosome. Both ALIX and ESCRT-I contain ubiquitin binding domains that facilitate this first step. Unlike ESCRT-I, ALIX possesses an ESCRT-III binding domain that allows for the direct recruitment of ESCRT-III and VPS4, the machinery required for sealing of cargo within intraluminal vesicles and abscising these vesicles from the endosomal membrane. In a similar fashion, the ESCRT-I component TSG101 binds ubiquitylated cargo, while the VPS28 component can recruit ESCRT-III through ESCRT-II which also binds ubiquitylated cargo as well as phospho-inositol lipids.
